# Potential Health Benefits of Dietary Antioxidants

**DOI:** 10.3390/antiox15010092

**Published:** 2026-01-11

**Authors:** Irene Dini

**Affiliations:** Department of Pharmacy, University of Naples Federico II, Via Domenico Montesano 49, 80131 Napoli, Italy; irdini@unina.it

Dietary antioxidants constitute a heterogeneous class of bioactive molecules, including polyphenols, vitamins, peptides, and specialized metabolites, that mitigate oxidative stress and its pathological consequences [1,2]. Excessive production of reactive oxygen species, whether arising from endogenous cellular metabolism or exogenous environmental exposures, contributes to the initiation and progression of chronic diseases such as cardiovascular dysfunction, neurodegeneration, metabolic disorders, inflammatory conditions, and cancer [3]. Antioxidants help maintain redox homeostasis and improve cellular adaptation to metabolic or inflammatory stress [4]. Numerous polyphenols, flavonoids, and sulfur-containing compounds activate Nrf2, promoting the transcription of detoxifying enzymes, antioxidant proteins, and stress-response mediators [5]. Compounds such as quercetin, resveratrol, catechins, and other phytochemicals have demonstrated therapeutic potential in chronic metabolic, cardiovascular, and degenerative diseases by attenuating NF-κB activation and reducing the expression of pro-inflammatory cytokines, adhesion molecules, and enzymes such as COX-2 and iNOS [6].

Moreover, several dietary antioxidants, including resveratrol, berberine, and alpha-lipoic acid, activate AMPK, an energy sensor that regulates glucose uptake, lipid oxidation, and mitochondrial biogenesis [7,8]. These mechanisms suggest their potential use in the treatment of obesity, type 2 diabetes, and non-alcoholic fatty liver disease, where metabolic imbalance and oxidative stress are tightly interconnected. Polyphenols such as resveratrol and anthocyanins enhance SIRT1 activity, promote mitochondrial biogenesis, improve oxidative phosphorylation efficiency, and support cellular persistence during metabolic stress [9], suggesting their potential for healthy longevity.

Curcumin, epigallocatechin gallate, and mangostin exert defensive effects against apoptosis, endothelial dysfunction, and metabolic imbalance by modulating the PI3K/Akt signaling cascade, a pathway central to cell survival, proliferation, and metabolic regulation [10,11,12].

As interest in functional foods and nutraceuticals continues to grow, the scientific community faces the dual challenge of elucidating the mechanisms underlying antioxidant activity and ensuring the safe, effective, and sustainable use of antioxidant-rich ingredients.

The volume Potential Health Benefits of Dietary Antioxidants presents a diverse collection of research and review articles that collectively deepen our understanding of antioxidant bioactivity, bioavailability, metabolic interactions, and translational potential. The contributions span molecular, cellular, animal, and human studies, supplying a multidimensional perspective on how dietary antioxidants influence health outcomes.

A central theme emerging from this collection is the application of advanced omics technologies to reveal the molecular processes by which antioxidants exert their effects.

Popescu et al. employed a data-independent acquisition proteomic approach to investigate how antioxidant supplementation modulates the cytoplasmic proteome of liver and kidney tissues in weaned piglets exposed to aflatoxin B1 and ochratoxin A. Their data show that antioxidants derived from grape seed and sea buckthorn can partially counteract mycotoxin-induced disruptions in metabolic pathways, oxidative stress responses, and detoxification processes. Notably, the study highlights organ-specific responses, underscoring the complexity of antioxidant–toxin interactions [13].

He et al. adopted an integrated strategy combining in vitro enzymology, untargeted metabolomics, and network pharmacology to explain the synergistic anti-diabetic effects of *Morus alba* and *Siraitia grosvenorii*. Their study shows that the combination more effectively inhibits carbohydrate-metabolizing enzymes, enhances antioxidant capacity, and modulates key signaling pathways involved in insulin regulation and inflammation. The identification of flavonoids, phenolic acids, and mogrosides as major contributors provides a mechanistic foundation for the development of synergistic functional food formulations [14].

Several contributions emphasize that antioxidant efficacy is controlled not only by molecular composition but also by individual genetic and bodily factors. Precision nutrition strategies may therefore optimize treatment results and reduce differences across clinical studies. The MiBlend randomized trial by DeBenedictis et al. examined how genetic polymorphisms influence individual responses to fruit- and vegetable-based interventions. Variants in genes such as *XRCC1* and *GSTP1* were associated with differential improvements in DNA damage resistance and microvascular function, suggesting that genetic stratification may increase the accuracy of dietary recommendations [15].

In the context of human health preservation, Zujko-Kowalska et al. demonstrated that dietary antioxidant quality, quantified using a newly developed Dietary Antioxidant Quality Index, significantly improves quality of life and reduces symptom severity in patients with rosacea undergoing cosmetic treatment. This work underscores the promise of dietary antioxidants as adjunctive therapies in chronic inflammatory skin conditions [16].

Chatatikun et al. conducted a systematic review and meta-analysis of the nephroprotective effects of alpha-mangostin, which can significantly reduce the markers of acute kidney injury, oxidative stress, and histopathological damage [17]. The meta-analytic approach strengthens the evidence by quantifying effect sizes and identifying consistent trends across multiple studies.

Synergistic interactions among antioxidant compounds demonstrate the improved value of whole-food matrices over isolated molecules.

Nina et al. provided an extensive chemical and functional characterization of South African herbal teas, identifying numerous phenolic compounds and demonstrating potent inhibitory activity against α-glucosidase and α-amylase, supporting their traditional use in glycemic control [18].

Acknowledging the need for environmentally responsible innovation, the volume also includes research on functional foods and the characterization of agri-food by-products rich in antioxidants, thereby valorizing materials that would otherwise be discarded.

Li et al. reviewed the antioxidant and anti-inflammatory properties of camel and donkey milk, which contain lactoferrin, lysozyme, immunoglobulins, and polyunsaturated fatty acids that modulate inflammatory pathways and oxidative stress, with prospective uses in diabetes management, nephroprotection, hepatoprotection, and immune regulation [19].

Dibwe et al. investigated extracts from *Allium cepa*. They identified specific fractions capable of reducing lipid droplet accumulation and oxidized lipid hydroperoxides in hepatocytes, showing the promise of *Allium*-derived ingredients in preventing metabolic dysfunction–associated fatty liver disease [20].

Alburquenque et al. developed flavonoid-enriched fractions from maqui and murta berries using preparative HPLC and demonstrated their anti-inflammatory properties in intestinal cell models. Chitosan-coated formulations demonstrated increased stability and bioactivity, suggesting a valuable role in the management of inflammatory bowel disease [21].

Perrone et al. explored the role of agri-food by-products within the Mediterranean diet as sustainable sources of bioactive compounds with anti-breast cancer potential, highlighting their ability to inhibit tumor proliferation, migration, and angiogenesis, and to enhance sensitivity to chemotherapy and radiotherapy [22].

Several challenges continue to limit the clinical translation of antioxidant research. The bioavailability and metabolic fate of many compounds remain poorly defined, particularly for polyphenols extensively transformed by the gut microbiota. Variability in botanical extracts and functional foods, driven by differences in cultivar, processing, and extraction methods, hampers standardization and reproducibility. Small sample sizes, short intervention periods, and heterogeneous populations often limit the scope of clinical studies. Moreover, the mechanistic complexity of multifunctional antioxidants, acting across ROS scavenging, enzyme modulation, gene regulation, mitochondrial pathways, and microbiome interactions, makes it difficult to identify primary modes of action. Microbiome-focused investigations remain limited despite their central relevance. Persistent discrepancies between in vitro, in vivo, and human findings underscore the need for integrated, harmonized research approaches that support safe, effective, and sustainable antioxidant-based strategies.

Upcoming investigations will be fundamental to advancing more effective, personalized, and environmentally responsible antioxidant-based strategies for the prevention and management of chronic and metabolic diseases.

Key priorities for future research should be to reduce metabolite and phytocomplex compositional variability and improve reproducibility across studies by developing and validating standardized analytical and manufacturing methods to obtain bioactives and phytocomplex; clarify the bioactive forms of antioxidant compounds improving in vivo and human investigations to elucidate the absorption, distribution, metabolism, and excretion pathways; and perform microbiome-focused studies to predict interindividual responses and optimize nutritional interventions, given the central role of the gut microbiota in modulating and activating many dietary antioxidants. Finally, rigorous, long-term, and well-controlled clinical trials are needed to substantiate the health benefits suggested by preclinical evidence and to translate mechanistic knowledge into meaningful outcomes.
